# Supplementary material for: Integration of single-cell transcriptomes and biological function reveals distinct behavioral patterns in bone marrow endothelium
Source: Nat Commun. 2022 Nov 24;13:7235. doi: 10.1038/s41467-022-34425-z (PMC9700769; doi:10.1038/s41467-022-34425-z)
Supplement: Supplementary file 1 — Supplementary Information [file 41467_2022_34425_MOESM1_ESM.pdf]

Supplementary Fig. 1

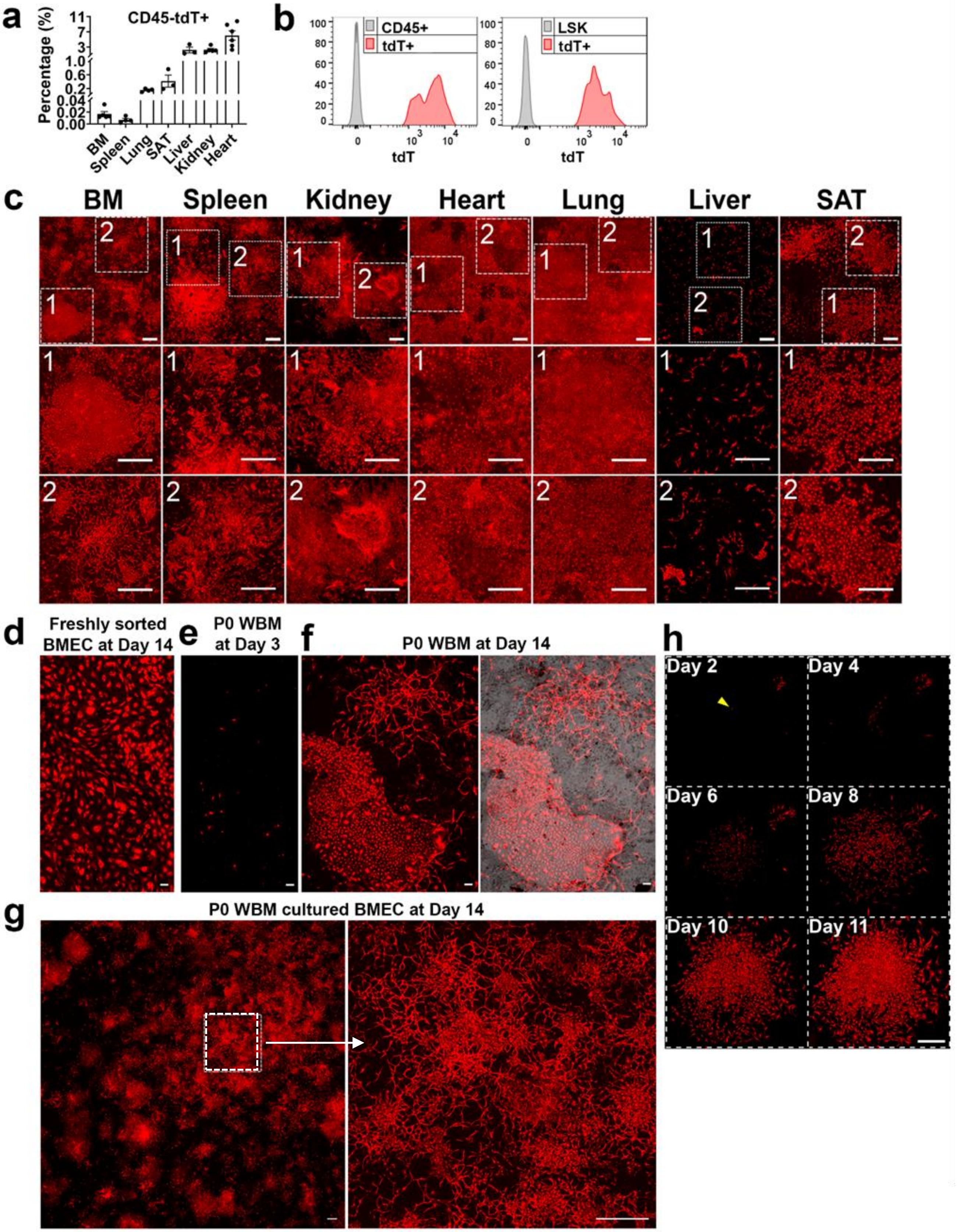

Supplementary Fig. 1. Endothelial cell-specific tdT reporter expression in *Tie2-CreERT2;Rosa26-tdTomato* mice in fresh and cultured tissues.

Analysis of 4-6-month-old *Tie2-CreERT2;Rosa26-tdTomato* mice induced with tamoxifen.

(a) Percentages of $CD45^{-}tdT^{+}$ cells in fresh tissues (T0). Heart n=6; BM n=5; Kidney n=5; Spleen n=4; Lung n=4; SAT n=3, and Liver n=3. Data are presented as the mean ± SEM. Source data are provided as a Source Data file.

(b) Since the transgenic *Tie2*-promoter could be activated in hematopoietic stem cells (HSC, $CD45^{+}$) labeling its progenies, $tdTomato^{+}$ expression was determined in BM blood cells. BM $CD45^{+}$ cells were gated and their tdT reporter expression was compared to gated $CD45^{-}tdT^{+}$ cells by flow cytometry (left histogram). BM LSK cells were gated and their tdT reporter expression was compared to gated $CD45^{-}tdT^{+}$ cells by flow cytometry (right histogram). Gating strategy is summarized in Suppl. Fig. 9a. Representative of n=4 independent experiments. Note that, tdTomato expression is not detected in the $CD45^{+}$ population, and $CD45^{+}$ primitive progenitors LSK ($Lin^{-}Sca\text{-}1^{+}c\text{-}Kit^{+}$) do not show presence of $tdTomato^{+}$ cells BM $CD45^{+}$ cells.

(c-h) Confocal microscopy. (c) Snapshot images of a large area (top) and two zoom-in images (white dashed square marked as 1 and 2) at day 12-14 of P0 culture of the indicated tissues. Scale bar, 1,000 μm. Representative of n=2-3 independent experiments.

(d-f) Snapshot images show tdT fluorescence of: (d) freshly sorted $CD45^{-}Ter119^{-}tdT^{+}$ BMEC at day 14 of culture; (e) tdT^{+} BMECs in P0 WBM culture at day 3; (f) tdT^{+} BMECs in P0 WBM culture at day 14 (left panel). In the right panel, the same snapshot image merged the tdT fluorescence with bright field to show tdT^{-} adherent cells filling empty areas surrounding tdT^{+} BMECs. Scale bar, 100 μm. Representative of n=5 independent experiments.

(g) Snapshot image of a large area (left) and zoom-in image (white dashed square projected in the right panel) at the day 14 of P0 WBM culture. Scale bar, 1,000 μm. Representative of n=7 independent experiments.

(h) Snapshot images from time-lapse imaging of P0 WBM culture at low cell density focused on single cell and its evolution at day 2, 4, 6, 8, 10, and 11. Note that a single tdT+ cell shows a clonal expansion. Scale bar, 1,000 μm. Representative of n=3 independent experiments.

Supplementary Fig. 2

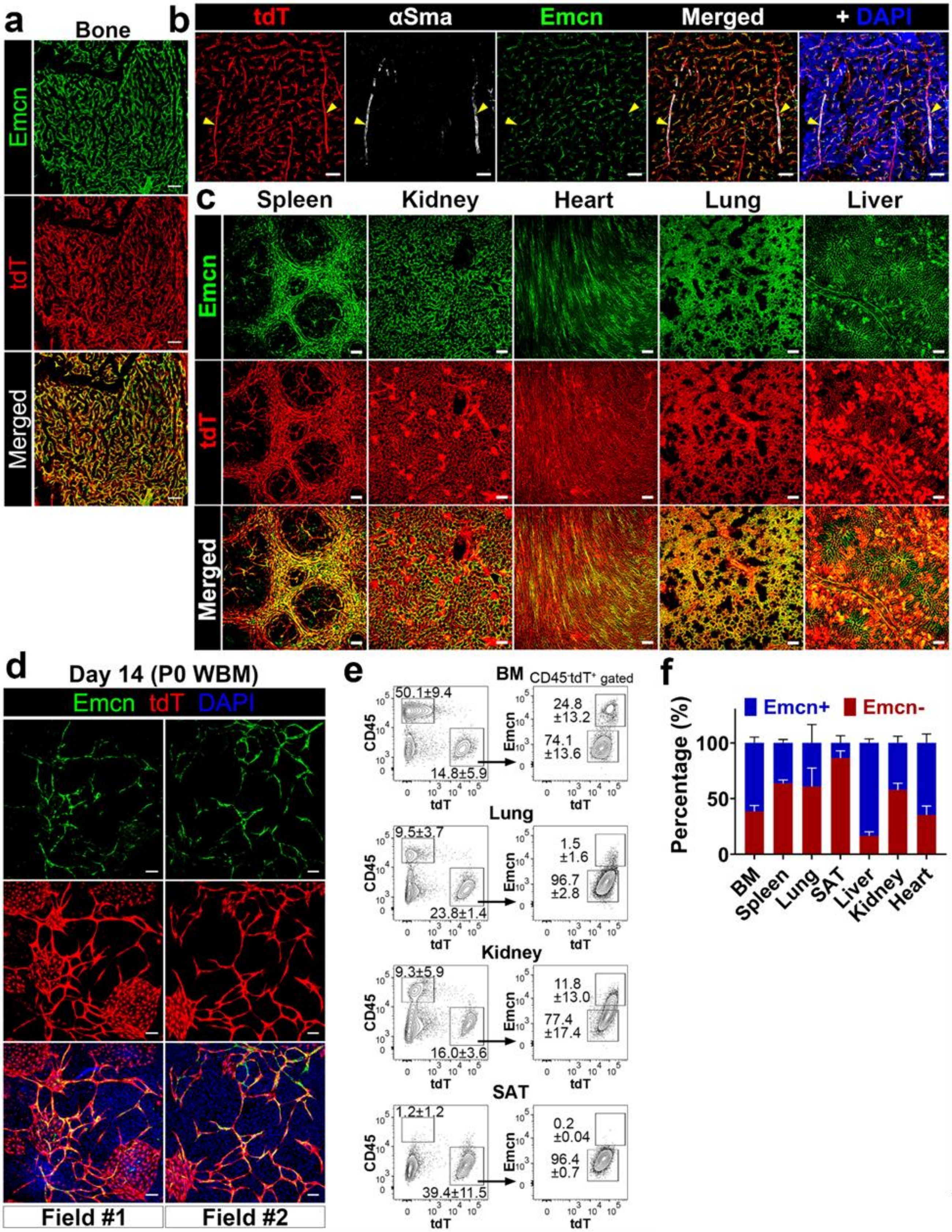

Supplementary Fig. 2. Differential expression of Emcn in vitro and in vivo.

(a-c) Confocal images detecting Emcn (in green) and αSma (in white) staining, and tdT reporter expression (in red): (a-b) in bone (scale bar, 200 μm (a); 100 μm (b)); (c) in the indicated tissues (scale bar, 100 μm) from 5-month-old *Tie2-CreERT2;Rosa26-tdTomato* mice. Representative of n=2 independent experiments.

(d) Network-forming BMECs express Emcn. Immunostaining for Emcn (in green) on tdT$^+$ BMECs in P0 WBM cultures at day 14. Confocal images show two fields in two independent cultures. Scale bar, 100 μm. Representative of n=5 experiments.

(e) Dot plots (left column) show distribution of tdT$^+$, Emcn$^+$ and Emcn$^-$ populations at the day 12-14 of P0 culture of the indicated tissue. *Emcn* expression on the gated tdT$^+$ ECs at day 12-14 of two independent P0 culture of the indicated tissue (n=2-3). Source data are provided as a Source Data file. Note the positive association between frequency of macrophages (which at day 14 represent the entirety of the CD45$^+$tdT$^-$ population) and frequency of CD45$^-$tdT$^+$Emcn$^+$ ECs in the various tissues (highest in the BM and lowest in SAT).

(f) Percentages of Emcn$^+$ and Emcn$^-$ cells in the CD45$^-$tdT$^+$ population at day 0 of the indicated tissue. BM n=3, Spleen n=2, Liver n=2, Kidney n=2, and Heart n=2; Lung n=2 and SAT n=2. Data are presented as the mean ± SEM.

Supplementary Fig. 3

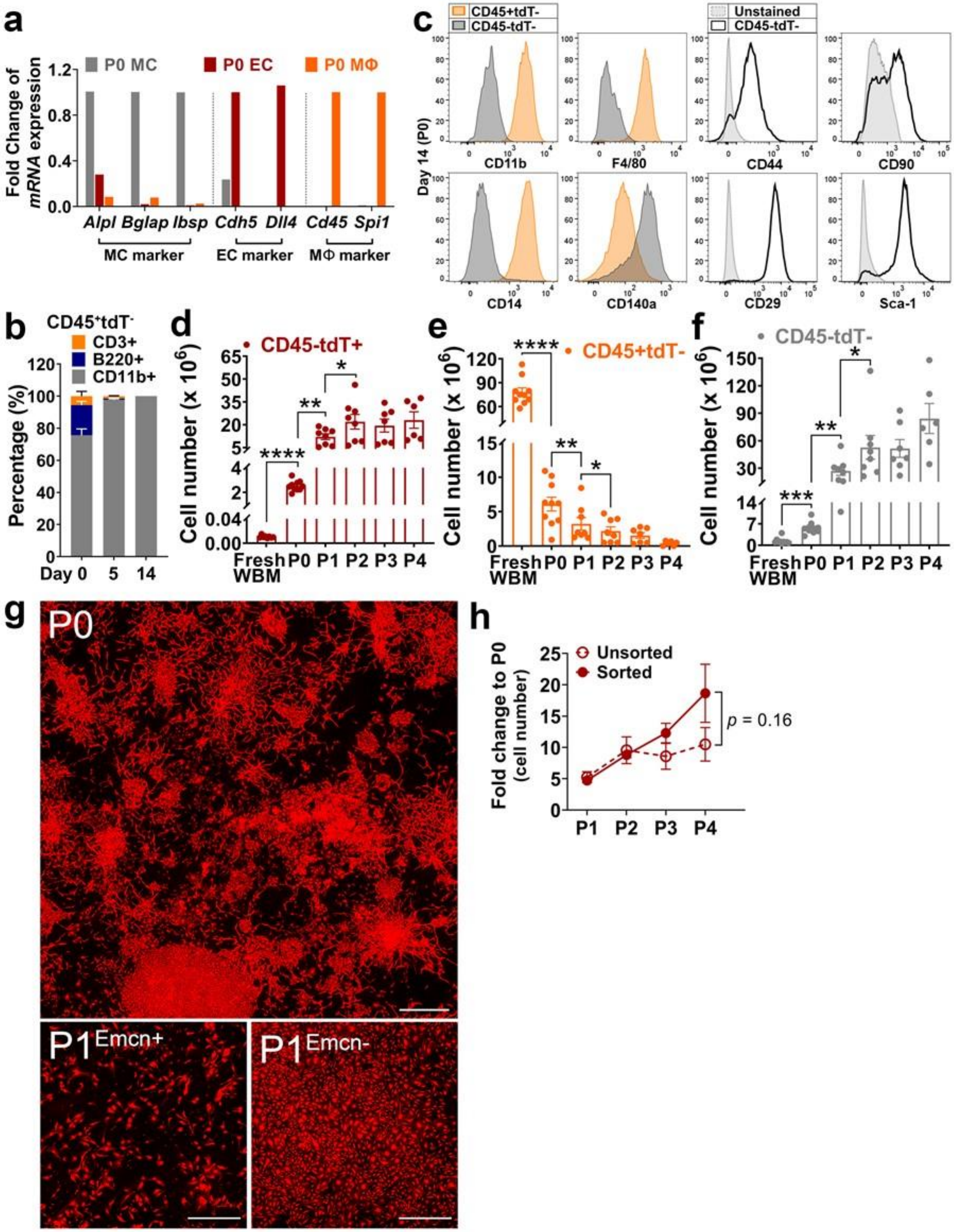

Supplementary Fig. 3. P0 WBM BMECs grow in the presence of MΦ and MC.

(a) P0 WBM cultures were harvested and the following populations: $CD45^{-}tdT^{-}$ (MC), $CD45^{-}tdT^{+}$ (EC), and $CD45^{+}tdT^{-}$ hematopoietic cells (MΦ), were sorted at day 14 for analysis of the indicated cell maker gene expression by qRT-PCR; n=1 (from 5 pooled mice). Source data are provided as a Source Data file.

(b) Relative percentages of $CD11b^{+}$ (gray), $B220^{+}$ (blue), and $CD3^{+}$ (orange) cells evaluated by flow cytometric analyses in total $CD45^{+}$ cells at: day 0, day 5 in WBM floating cells, and day 14 in WBM adherent cells. Day 0 n=4; Day 14 n=4; Day 5 n=2. Data are presented as the mean ± SEM. Source data are provided as a Source Data file.

(c) Expression of myeloid markers CD11b and CD14, macrophage marker F4/80 and mesenchymal marker CD140a on gated $CD45^{+}tdT^{-}$ cells (orange) and $CD45^{-}tdT^{-}$ cells (gray) at day14 of P0 WBM culture (Left panels). Additional analysis (Right panels) of expression of mesenchymal markers CD44, CD90, CD29, and Sca-1 on gated $CD45^{-}tdT^{-}$ cells (black solid line) compared to unstained controls (gray). Representative of n=4 independent experiments.

(d-f) P0 WBM cultures were harvested and serially passaged up to 4 times (P4). At each passage, the absolute numbers of (d) $CD45^{-}$ tdT^{+} (red), (e) $CD45^{+}tdT^{-}$ (orange), and (f) $CD45^{-}$ tdT^{-} (gray), populations were evaluated by flow cytometry and cell counting. $CD45^{-}tdT^{+}$ Fresh WBM n=8; $CD45^{-}tdT^{+}$ P0 n=10; $CD45^{-}tdT^{+}$ P1-2 n=8; $CD45^{-}tdT^{+}$ P3 n=7. // $CD45^{+}tdT^{-}$ Fresh WBM n=10; $CD45^{+}tdT^{-}$ P0 n=10; $CD45^{+}tdT^{-}$ P1-2 n=8; $CD45^{+}tdT^{-}$ P3 n=7; $CD45^{+}tdT^{-}$ P4 n=6. // $CD45^{-}tdT^{-}$ Fresh WBM n=10; $CD45^{-}tdT^{-}$ P0 n=10; $CD45^{-}tdT^{-}$ P1-2 n=8; $CD45^{-}tdT^{-}$ P3 n=7; $CD45^{-}tdT^{+}$ P4 n=6. Data are presented as the mean ± SEM. Statistics for all comparisons shown were determined using paired t-test, two-sided. (d) Fresh WBM vs. P0, $p < 0.0001$; P0 vs. P1, p 0.0015; P1 vs. P2, p 0.0158; (e) Fresh WBM vs. P0, $p < 0.0001$; P0 vs. P1, p = 0.0029; P1 vs. P2, p 0.0471; (f) Fresh WBM vs. P0, $p = 0.0005$; P0 vs. P1, $p = 0.0013$; P1 vs. P2, $p = 0.0180$. *p < 0.05; **p < 0.01; ***p < 0.001. Source data are provided as a Source Data file.

(g) Snapshot image of P0 WBM culture at day 14 (top), and images of the derived P1 cultures of sorted $Emcn^{+}$ (bottom left) and $Emcn^{-}$ (bottom right) at day 7. Scale bar, 1,000 μm. Representative of n=4 experiments. Note the lack of 2D-networks in $P1^{Emcn+}$ culture.

(h) Line graph shows fold change of the $Emcn^{-}$ cell numbers at the end of each passage compared to the input cell number at P0 in two conditions: sorted cell cultures ($P1^{Emcn-}$ to $P4^{Emcn-}$) vs. unsorted WBM cultures (P1 to P4). Unsorted P1 and P2 n=8 (each group), Sorted P1, P2, and P3 n=8 (each group); Unsorted P3 n=7; Unsorted P4 and Sorted P4 n=6 (each). Data are presented as the mean ± SEM. Source data are provided as a Source Data file.

Supplementary Fig. 4

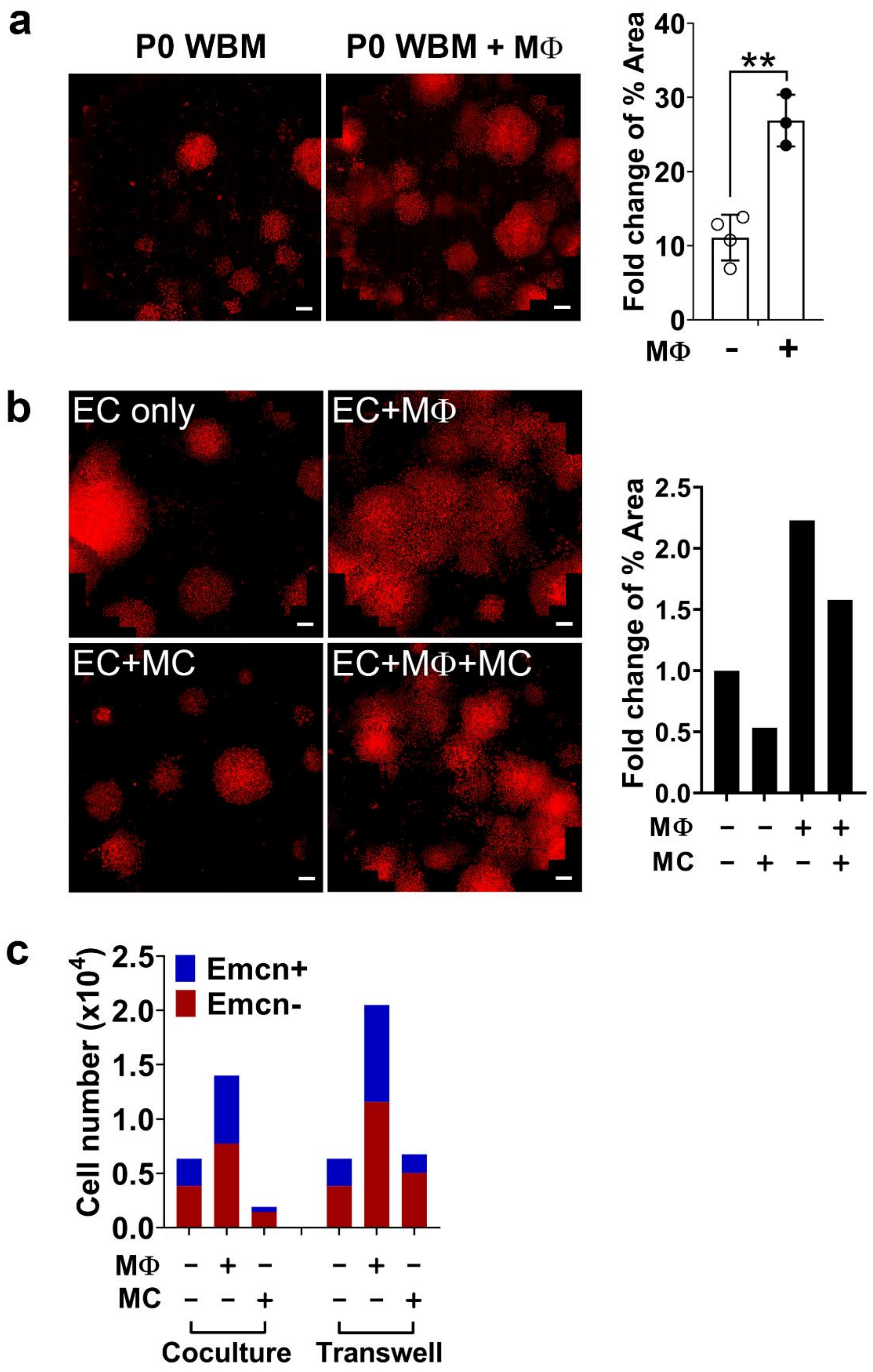

Supplementary Fig. 4. Macrophages and mesenchymal cells affect BMEC growth in opposite ways in ex vivo culture.

(a) Snapshot images at day 14 culture showing tdT^+ BMECs in P0 WBM cultures that were supplemented with MΦ compared to control. Scale bar, 2,000 μm. Representative of n=3-4 independent experiments. On the right, the bar graph shows quantification of tdTomato fluorescence (BMEC) in each condition by ImageJ. Values indicate fold change in percentage of area covered by BMEC over the entire area of the well. P0 WBM n=4; P0 WBM + MΦ n=3. Statistics were determined using two-sided paired T test. p = 0.0014. Source data are provided as a Source Data file.

(b) $CD45^-tdT^+$ (BMEC), $CD45^+tdT^-$ (HC/MΦ), and $CD45^-tdT^-$ (MC) populations were sorted from fresh BM and mixed in different combination maintaining the same relative ratio observed in BM in vivo (BMEC):(HC/MΦ):(MC) = 1:4,000:5,300. Admixed populations were cultured in the same conditions as WBM for 17 days. On the left, snapshot images of cultures at day 17 show BMEC growth in the different combinations; top left: sorted BMEC alone (control); bottom left: BMEC+MC; top right: BMEC+HC/MΦ; bottom right: BMEC+MC+HC/MΦ. Each square is the image of an entire well. Scale bar, 2,000 μm. On the right, the bar graph shows quantification of tdTomato fluorescence (BMEC) in each condition by ImageJ. Values indicate fold change in percentage of area covered by BMEC over the entire area of the well. n=1 supporting 8 independent experiments in Fig. 5a-d. Source data are provided as a Source Data file.

(c) Total cell number of $Emcn^+$ and $Emcn^-$ cells of P0 WBM culture with or without additional HC/MΦ or MC by coculture (left) or transwell (right) system. Representative of n=3 independent experiments.

Supplementary Fig. 5

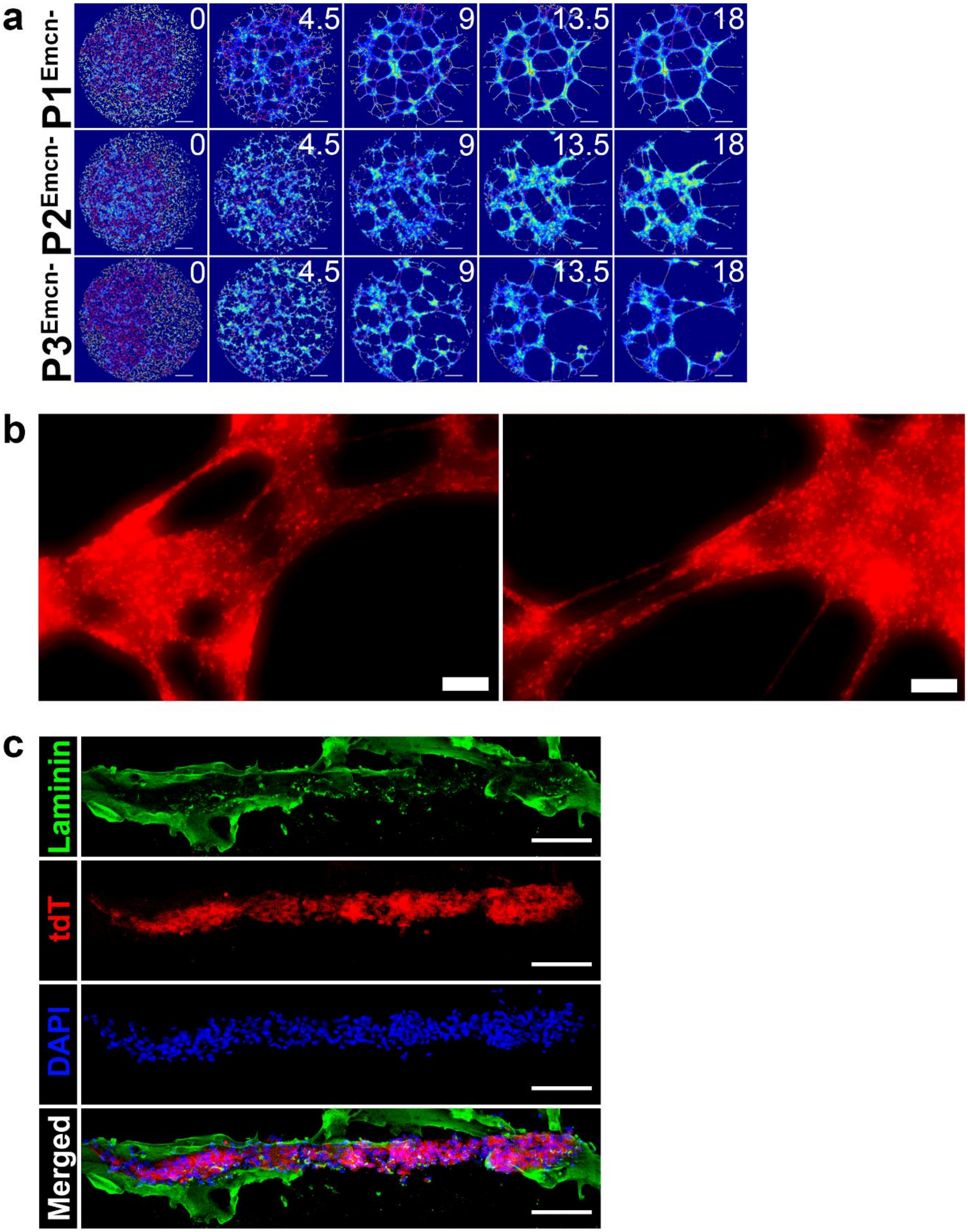

Supplementary Fig. 5. Kinetics of cord-network formation by Emcn^{-} BMECs after P0 culture.

(a) Skeletonized images from time lapse microscopy of Matrigel tube formation by Emcn^{-} cells from P1, P2, and P3 cultures (grown as sorted cells) at the indicated time-points during 18 hours from seeding. Original images for all observation times underwent contrast-limited adaptive histogram equalization, cropping of the growth boundary, and gaussian blurring. Following this, images were converted to binary and skeletonized and used for quantification. Representative of n=12 experiments.

(b) Snapshot images with high magnification of Matrigel tube formation assay using P0 Emcn^{-} cells at 20 hours after plating. Scale bar, 200 μm. Representative of n>20 independent experiments.

(c) Immunostaining of coronally sectioned structures formed on the Matrigel using P0 Emcn^{-} cells with antibody for Laminin β-1. Scale bar, 100 μm. Representative of n=8 independent images. Note the absence of lumen. We refer to these structures as “cords”. These types of structures are commonly referred in literature as “tubes” even in the absence of lumen in recognition of cells’ ability to engage in the morphogenesis process within the limitations of current angiogenesis assays.

Supplementary Fig. 6

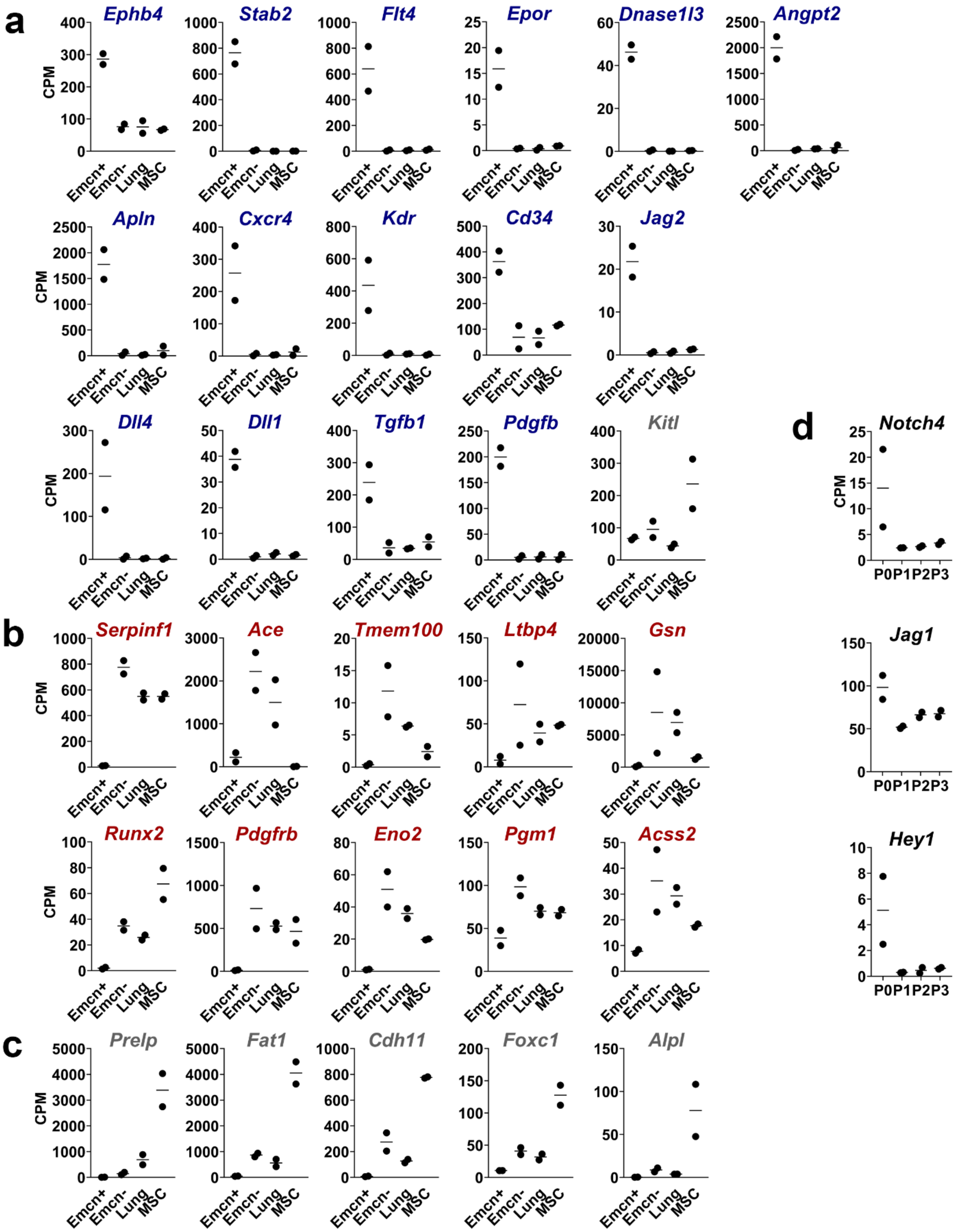

Supplementary Fig. 6. Representative differentially expressed genes in Emcn$^+$ and Emcn$^-$ BMECs, lung ECs, and MSC at P0 culture.

(a-c) Bulk-RNA-seq of sorted populations at day 14 of culture: Emcn$^+$ and Emcn$^-$ BMEC from P0 WBM; Emcn$^-$ EC from lung; MSC from BM and MSC (Osx-Cre-derived tdT$^+$ MSC; see methods). Cluster Graphs show expressions across these four populations of some of the genes highly expressed in: (a) sinusoidal EC; (b) arterial EC; and (c) MSC; expression is indicated in count per million (CPM). Source data are provided as a Source Data file.

(d) Cluster Graphs show expression of Notch pathways genes in Emcn$^-$ BMECs across passages; expression is indicated in CPM. Source data are provided as a Source Data file.

Supplementary Fig. 7

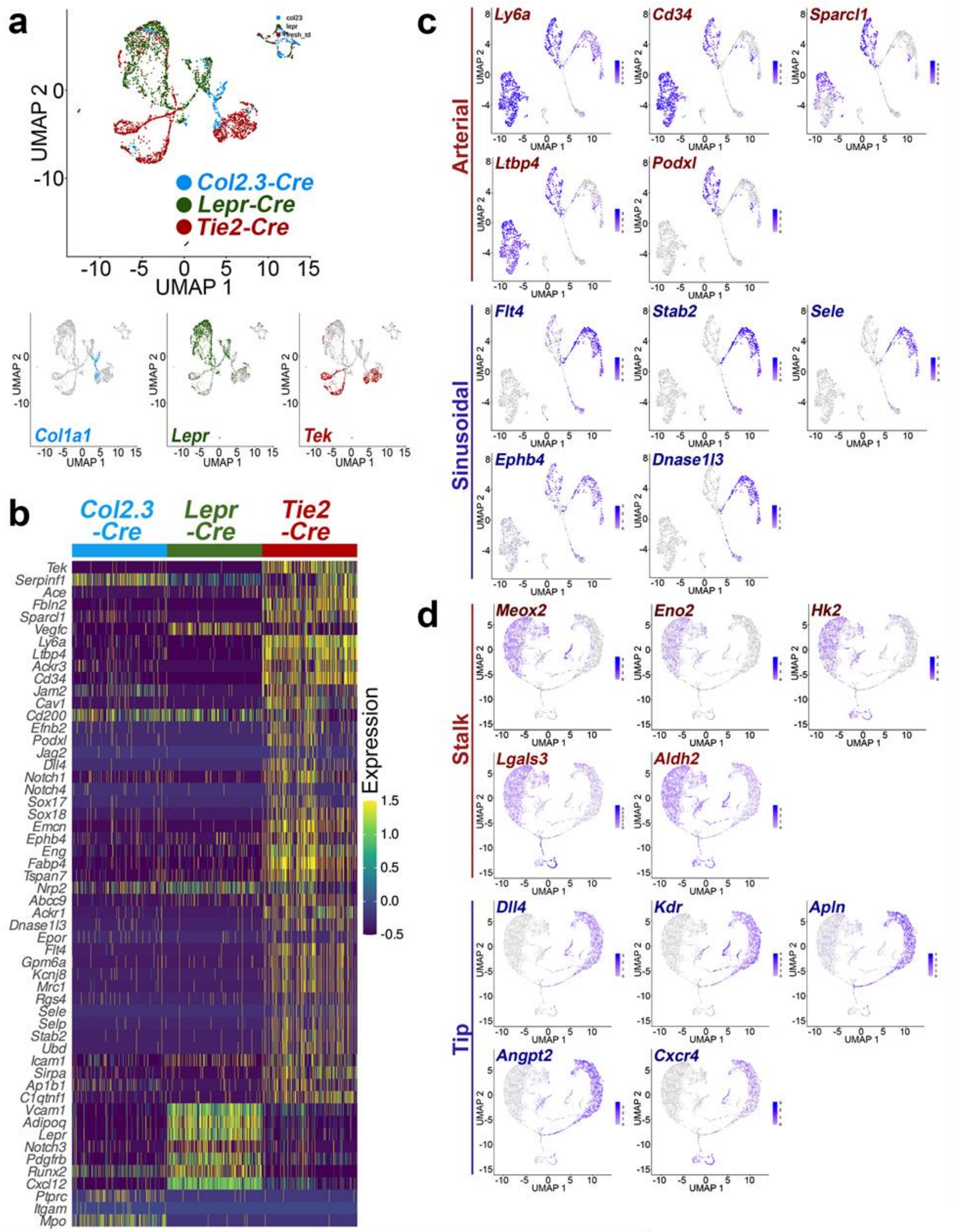

Supplementary Fig. 7. scRNA-seq analysis in fresh and cultured BMEC.

(a) UMAP plots of single-cell gene expression profile from fresh Tie2Cre tdT$^+$ cells sorted from BM and integrated with publicly available datasets from sorted BM LeprCre tdT$^+$ and Col2.3Cre tdT$^+$ cells, as reported by Tikhonova and coworkers. Simultaneous (top) and single (bottom) visualizations of Tie2Cre, LeprCre, and Col2.3Cre populations.

(b) Gene expression analysis of endothelial, perivascular, and hematopoietic genes in endothelial Tie2Cre tdT$^+$, perivascular LepRCre tdT$^+$, and osteoblastic-specific Col2.3CretdT$^+$ cells. Clustering of cells using PCA and a nearest neighbor approach showed three separate populations. Moreover, gene expression of the endothelial signature was evident in Tie2Cre cells, while the perivascular signature was expressed by Lepr-Cre cells.

(c) UMAP plots of gene expression of arterial/sinusoidal signature genes in fresh sorted BMEC (Tie2Cre tdT$^+$ cells).

(d) UMAP plots of gene expression of tip/stalk signature genes in cultured WBM BMEC (Tie2Cre tdT$^+$ cells) sorted at P0. Note that the right cluster is identified by *Endomucin* expression (Fig. 8f).

Supplementary Fig. 8

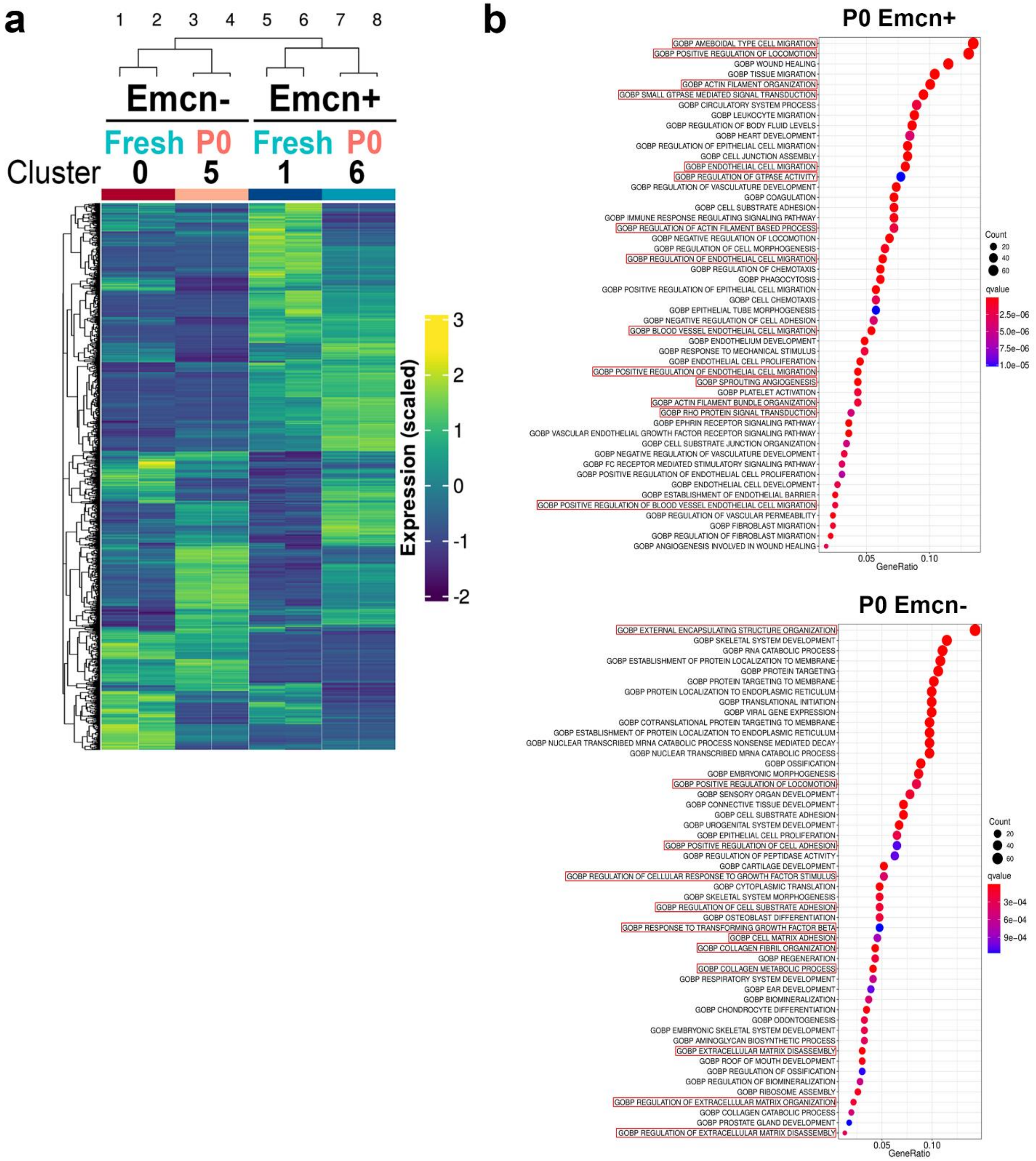

Supplementary Fig. 8. Differentially expressed genes and pathways enrichment in Emcn$^+$ and Emcn$^-$ BMECs.

(a) Heatmap of the top 5% variable genes from single cell gene expression profiles in fresh and cultured BMECs (Tie2Cre tdT$^+$ cells freshly sorted from BM, and cultured Tie2Cre tdT$^+$ cells sorted at WBM P0, respectively). The Dendrogram above heatmap shows hierarchical clusters and the distance between the subpopulation's transcriptomes. The analysis was carried out by first building a distance matrix between the subpopulation's transcriptome set using Euclidian distance and the Ward method to perform the hierarchical clusters and build the dendrogram. The results show distinct differences between Emcn$^-$ and Emcn$^+$ and the linkage between freshly isolated and cultured cells of the respective groups. The levels of expression are reported as z-scaled $\log 2$ counts per million (CPM).

(b) GO BP (Gene Ontology Biological Process) analysis ranked by GeneRatio from P0 Emcn$^+$ (top) and P0 Emcn$^-$ (bottom).

Supplementary Fig. 9

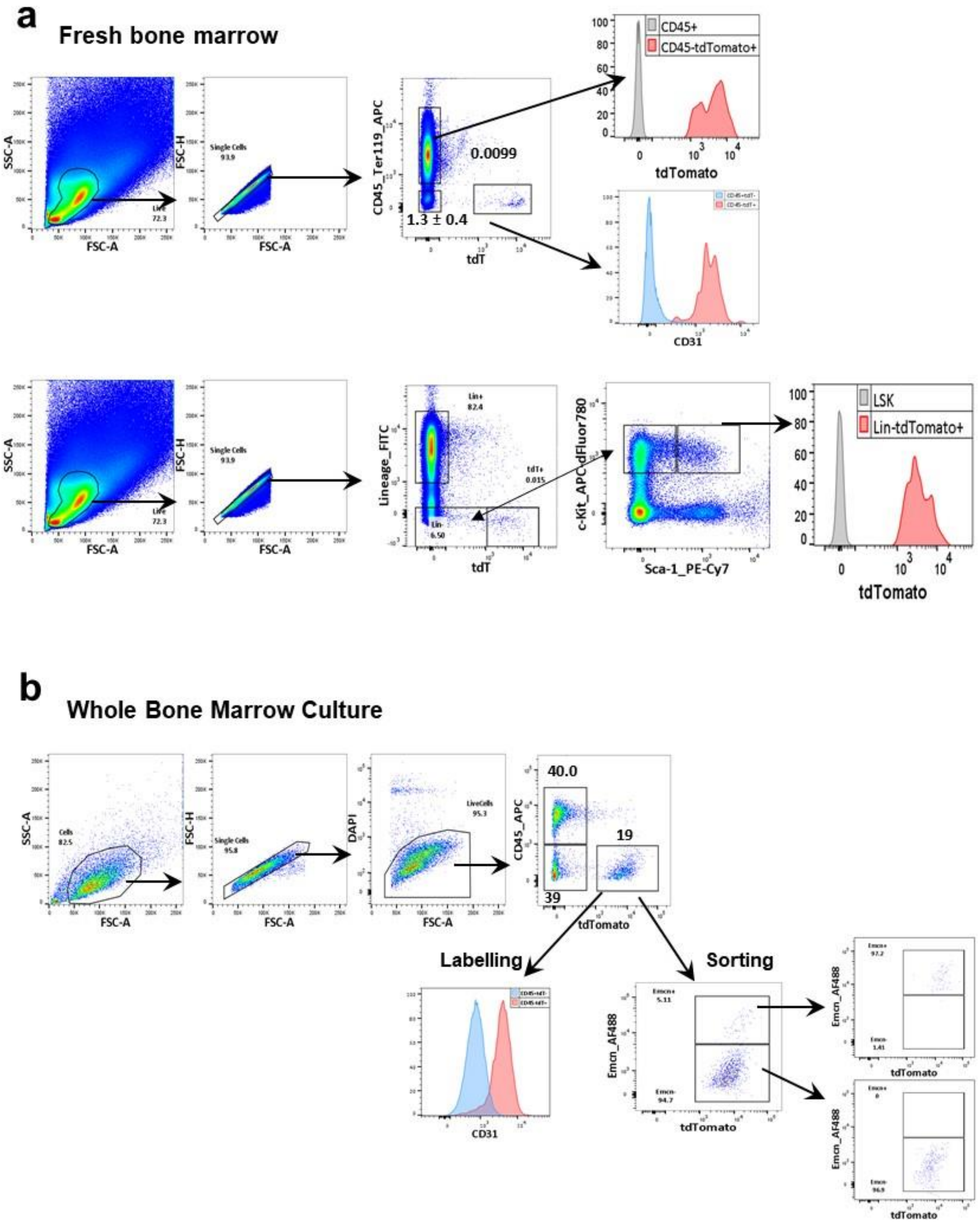

Supplementary Fig. 9. Gating Strategy.

(a) Fresh Bone Marrow. Gating strategy used for identifying and characterizing dT^+ cells from fresh BM of 4-6-month-old *Tie2-CreERT2;Rosa26-tdTomato* mice. Upper plots: viable single cells (DAPI negative) were gated to identify CD45 and Ter119 negative cells; $CD45^-Ter119^-$ were gated to identify tdT^+ cells. Gated $CD45^-Ter119^-$ tdT^+ cells were analyzed for expression of various endothelial markers, i.e.CD31 in the histogram, like shown in Figs. 1b and c, or sorted for experiments shown in Figs. 2a and c and Fig.8. Histogram shows levels of tdTomato expression on gated $CD45^+$; $CD45^-tdT^+$ cells were used as internal positive control.

Lower plots: viable single cells and DAPI negative cells were gated to identify Lineage negative cells. $Lin^-Sca1^+c\text{-}Kit^+$ cells (LSK) could be identified only on the Lin^-tdT^- fraction (and viceversa). Histogram shows levels of tdTomato expression on gated LSK; Lin^-tdT^+ gated cells were used as internal positive control (this gating was used in Suppl. Fig.1b)

(b) Whole bone marrow culture (day 14). Viable single cells and DAPI negative cells were gated to identify CD45 negative cells. Dot plot shows $CD45^+$ and $CD45^-$ fractions and clearly identifies the $CD45^-tdT^+$ population. $CD45^-tdT^+$ cells were gated to analyze the expression of various endothelial markers, i.e.CD31 in the histogram, and expression of Endomucin for sorting of $Emcn^+$ and $Emcn^-$ BMECs. This gating strategy was used in Figs. 2d, 3d, 3e, 4a and for Suppl. Figs. 2e, 3c, 4e, 4f.

Supplementary Table 1

# Mouse (4-6-month-old)	CD45-Ter119- tdT+ % in BM	CD45-Ter119- tdT+Emcn+ % in BM	CD45-Ter119- tdT+Emcn- % in BM
#1	0.0121	0.0074	0.0047
#2	0.0150	0.0081	0.0069
#3	0.0141	0.0076	0.0065
#4	0.0141	0.0078	0.0063
#5	0.0201	0.0131	0.0070
#6	0.0131	0.0074	0.0057
#7	0.0101	0.0068	0.0032
#8	0.0192	0.0142	0.0049
#9	0.0105	0.0072	0.0033
#10	0.0079	0.0034	0.0044
#11	0.0015	0.0007	0.0009
#12	0.0050	0.0023	0.0027
#13	0.0205	0.0157	0.0048
#14	0.0080	0.0058	0.0023
#15	0.0049	0.0024	0.0025
#16	0.0056	0.0024	0.0032
#17	0.0031	0.0014	0.0017
#18	0.0200	0.0125	0.0075
#19	0.0110	0.0056	0.0054
#20	0.0140	0.0069	0.0071
#21	0.0170	0.0091	0.0079
#22	0.0110	0.0070	0.0040
#23	0.0097	0.0062	0.0035
#24	0.0097	0.0062	0.0035
**Mean±SD**	**0.0116 ± 0.0054**	**0.0070 ± 0.0039**	**0.0046 ± 0.0020**

Supplementary Table 1. Summary of BMECs frequencies in fresh BM.

Percentages of CD45$^-$Ter119$^-$tdT$^+$ (left column, in black), CD45$^-$Ter119$^-$tdT$^+$Emcn$^+$ (middle column, in blue), and CD45$^-$Ter119$^-$tdT$^+$Emcn$^-$ (right column, in red) BMECs in total BM. BMEC were freshly isolated from tibia and femur of both legs of 4-6-month-old *Tie2-CreERT2;Rosa26-tdTomato* mice. Percentages were determined by flow cytometry analysis.

Supplementary Table 2

# Mouse (4-6-month-old)	# sorted CD45-Ter119-tdT+ from fresh BM	# sorted CD45-tdT+ T0^{tdT+} culture (Day 14) (x 10^4)	fold of proliferation (Day14 / Day0)
**#1**	4,345	2.4	5.5
**#2**	6,255	2.6	4.2
**#3**	8,092	8.4	10.4
**#4**	8,818	7.0	7.9
**Mean±SD**	**6,877 ± 2,003**	**5.1 ± 3.0**	**7.0 ± 2.7**

Supplementary Table 2. Summary of absolute BMEC numbers at time of sorting and after T0^{tdT+} culture.

BMEC proliferation capacity in T0^{tdT+} culture. Absolute numbers of CD45^{-}Ter119^{-}tdT^{+} BMECs freshly sorted from tibia and femur of both legs of 4 to 6-month-old *Tie2-CreERT2;Rosa26-tdTomato* mice (left column) and after T0^{tdT+} culture at day 14 (middle column). Fold increase in cell number after culture (right column). All numbers were calculated by flow cytometry analysis and manual cell counting (n=4).

Supplementary Table 3

# Mouse (4-6-month-old)	T0 Fresh CD45-Ter119-tdT+ # per mouse (x 10^3)	P0 WBM day14 CD45-Ter119-tdT+ % per mouse	P0 WBM day14 CD45-Ter119-tdT+ # per mouse (x 10^6)	fold of proliferation (Day14 / Day0)
**#1**	9.78	16.18	2.25	229.69
**#2**	11.42	18.32	2.50	219.23
**#3**	9.20	11.48	2.62	285.06
**#4**	9.90	15.35	2.89	291.78
**#5**	15.14	12.58	2.23	147.42
**#6**	9.13	33.32	3.39	371.51
**#7**	8.13	20.66	2.29	281.85
**#8**	11.27	22.80	2.79	247.40
**Mean±SD**	**10.49 ± 2.17**	**18.84 ± 6.99**	**2.62 ± 0.40**	**259.24 ± 65.42**

Supplementary Table 3. Summary of absolute BMEC numbers after P0 WBM culture.

BMEC proliferation capacity in P0 WBM culture. Absolute numbers of $CD45^-Ter119^-tdT^+$ BMECs within WBM at seeding (T0; first column). Percentages and absolute numbers of $CD45^-tdT^+$ BMECs after culture in P0 WBM day 14 (second and third column). Fold increase in cell number after culture (fourth column). All numbers were calculated by determining percentages by flow cytometry analysis and manual cell counting of total WBM cells; (n=8).

Supplementary Table 4

# Mouse (4-6-month-old)	T0 Fresh CD45-Ter119-tdT+Emcn+ # per mouse (x 10^3)	P0 WBM day14 CD45-Ter119-tdT+ Emcn+ % per mouse	P0 WBM day14 CD45-Ter119-tdT+ Emcn+ # per mouse (x 10^6)	fold of proliferation (Day14 / Day0)
#1	5.94	1.63	0.22	37.84
#2	6.10	0.65	0.09	14.45
#3	4.97	1.59	0.36	72.85
#4	5.46	1.24	0.23	42.66
#5	9.90	0.77	0.14	13.71
#6	5.13	4.93	0.50	97.17
#7	5.54	3.53	0.39	70.36
#8	8.18	2.56	0.31	38.17
**Mean±SD**	**6.40 ± 1.73**	**2.11 ± 1.48**	**0.28 ± 0.14**	**48.40 ± 29.43**
**# Mouse (4-6-month-old)**	**T0 Fresh CD45-Ter119-tdT+Emcn- # per mouse (x 10^3)**	**P0 WBM day14 CD45-Ter119-tdT+ Emcn- % per mouse**	**P0 WBM day14 CD45-Ter119 tdT+Emcn- # per mouse (x 10^6)**	**fold of proliferation (Day14 / Day0)**
#1	3.77	14.56	2.01	533.96
#2	5.15	17.68	2.41	467.62
#3	4.23	9.89	2.25	532.32
#4	4.43	14.11	2.65	597.23
#5	5.24	11.81	2.09	399.66
#6	3.92	28.40	2.87	731.79
#7	2.59	17.13	1.89	728.91
#8	2.84	20.24	2.47	868.86
**Mean±SD**	**4.02 ± 0.96**	**16.73 ± 5.76**	**2.33 ± 0.33**	**607.54 ± 156.95**

Supplementary Table 4. Summary of absolute BMEC Emcn$^+$ and Emcn$^-$ numbers after P0 WBM culture.

BMEC Emcn$^+$ and Emcn$^-$ proliferation capacity in P0 WBM culture. Absolute numbers of CD45$^-$Ter119$^-$ tdT$^+$ BMECs Emcn+ (blue) and Emcn$^-$ (red) at seeding (T0; first column). Percentages and absolute numbers of BMECs Emcn$^+$ and Emcn$^-$ after culture in P0 WBM day 14 (second and third column). Fold increase in cell number after culture (fourth column). All numbers were calculated by determining percentages by flow cytometry analysis and manual cell counting of total WBM cells; (n=8).

Supplementary Table 5

gene symbol	log2Fold Change (P0Emcn+ / P0Emcn-)	P0 Emcn+_1 (log2CPM)	P0 Emcn+_2 (log2CPM)	P0 Emcn-_1 (log2CPM)	P0 Emcn-_2 (log2CPM)	p value	p value adjusted	signifi -cance
Ace	-3.1802	114.4315	339.3165	1980.6486	2886.0410	8.58E-08	7.06E-07	TRUE
Acss2	-2.2023	6.9400	8.6568	52.4940	24.9127	1.41E-12	1.99E-11	TRUE
Aldh2	-1.8269	70.2561	99.8626	244.6700	406.3696	2.65E-13	4.02E-12	TRUE
Alpl	-4.6724	0.4120	0.1932	7.1754	11.9016	1.69E-18	3.97E-17	TRUE
Angpt2	6.0305	1769.2682	2291.2751	28.7521	6.5560	3.47E-11	4.24E-10	TRUE
Apln	4.5375	2046.3630	1534.7694	83.4869	9.7835	2.58E-06	1.71E-05	TRUE
Cd34	2.1674	319.0837	417.3825	26.7631	123.3532	1.89E-05	1.08E-04	TRUE
Cdh11	-5.1039	9.9506	6.0675	227.5496	375.2035	1.38E-70	4.37E-68	TRUE
Cdh5	5.1929	6222.4646	5118.1138	119.4900	6.3543	2.46E-06	1.63E-05	TRUE
Cxcl12	-2.6088	217.0745	406.6387	2541.0129	1857.7616	5.96E-07	4.39E-06	TRUE
Cxcr4	4.8832	339.2700	178.4310	8.9882	0.9078	6.41E-07	4.68E-06	TRUE
Dll1	5.0275	35.3657	43.2455	1.5862	0.4034	1.02E-23	3.65E-22	TRUE
Dll4	4.1955	270.0914	119.4178	8.4091	0.4034	1.29E-04	6.23E-04	TRUE
Dnase1l3	6.3447	42.5910	51.2453	0.7050	0.0000	3.08E-20	8.53E-19	TRUE
Efnb2	3.2384	218.1203	125.1761	20.1416	16.1378	3.29E-16	6.40E-15	TRUE
Emcn	5.3897	630.8470	797.6643	12.6640	2.5215	3.52E-07	2.68E-06	TRUE
Eno2	-5.6176	1.2042	0.8502	68.8339	43.2694	1.65E-75	6.44E-73	TRUE
Ephb4	1.8126	300.5135	278.7574	74.8512	91.4811	6.26E-11	7.47E-10	TRUE
Epor	4.9425	19.2357	12.6761	0.4532	0.3026	1.10E-10	1.28E-09	TRUE
Esm1	3.7141	368.6464	52.7139	6.6971	11.8008	1.47E-04	7.01E-04	TRUE
Fat1	-4.1701	43.9853	54.8781	1043.6366	867.2042	2.35E-67	6.68E-65	TRUE
Fbln2	-5.8137	31.3094	50.3564	3230.1071	1987.3681	3.34E-55	5.54E-53	TRUE
Flt4	6.2335	807.4855	482.5792	11.8332	1.3112	2.75E-20	7.64E-19	TRUE
Foxc1	-1.9473	10.6161	10.8210	39.4020	50.2289	6.76E-13	9.81E-12	TRUE
Gsn	-5.2327	67.7843	302.9115	2429.9068	16076.6625	3.12E-15	5.65E-14	TRUE
Hk2	-1.9511	13.9118	8.8501	72.1321	28.9472	1.52E-06	1.05E-05	TRUE
Il6st	-1.8242	291.6087	473.1108	1534.8397	1435.4559	5.99E-10	6.51E-09	TRUE
Jag1	0.5575	167.7653	181.6000	93.7843	121.6385	1.78E-02	4.93E-02	FALSE
Jag2	5.0428	25.0982	18.7049	0.7805	0.3026	1.01E-24	3.84E-23	TRUE
Kdr	5.0417	587.0836	287.9166	14.8544	1.8155	3.08E-08	2.71E-07	TRUE
Lgals3	-1.2003	59.7034	41.2745	200.4844	84.1182	1.84E-04	8.63E-04	FALSE
Ltbp4	-2.9678	3.2640	12.4828	27.8961	129.7074	2.21E-05	1.25E-04	TRUE
Ly6a	-1.9240	63.7914	268.7479	604.3736	874.4662	1.16E-05	6.86E-05	TRUE
Meox2	-1.8620	1.7112	19.3619	36.0283	78.6717	1.71E-03	6.40E-03	TRUE
Mrc1	3.1660	395.8995	236.5167	40.8874	29.0480	6.09E-14	9.87E-13	TRUE
Pdgfb	3.3158	215.9337	187.8994	9.2148	1.8155	1.67E-03	6.27E-03	TRUE
Pdgfra	-6.2004	14.3871	18.5503	1146.7112	1565.7685	6.24E-76	2.53E-73	TRUE
Pdgfrb	-5.7662	16.2251	9.8162	1077.1472	536.6822	5.11E-55	8.32E-53	TRUE
Pecam1	5.8160	2473.9839	2263.7202	47.8111	5.1439	1.60E-11	2.04E-10	TRUE
Pgm1	-1.3323	47.2810	30.7626	120.9251	95.3138	5.66E-10	6.17E-09	FALSE
Podxl	4.3901	34.8587	27.5936	1.8631	0.1009	5.37E-07	3.98E-06	TRUE
Prelp	-4.1326	5.6091	11.1688	114.0769	212.6153	4.43E-26	1.84E-24	TRUE
Runx2	-4.1388	2.5669	1.3913	42.2973	34.0911	8.52E-46	9.96E-44	TRUE
Sele	4.8173	1.3943	6.2607	0.0504	0.0000	5.16E-05	2.72E-04	TRUE
Selp	6.3658	12.5174	61.4480	0.3273	0.1009	5.32E-12	7.12E-11	TRUE
Serpinf1	-6.1224	9.9189	12.9466	921.2261	784.8006	2.32E-213	8.83E-210	TRUE
Sox17	2.8199	213.5253	112.5000	4.9850	0.2017	4.58E-03	1.53E-02	TRUE
Sparcl1	-2.3594	177.2405	158.1416	618.3217	1457.5445	2.19E-05	1.24E-04	TRUE
Stab2	6.6015	844.8160	701.6277	11.0779	1.9164	4.30E-29	2.15E-27	TRUE
Tek	2.0620	168.3357	124.5964	20.3178	49.4220	1.81E-08	1.64E-07	TRUE
Tmem100	-5.0324	0.4753	0.1546	8.6357	17.0455	8.46E-23	2.82E-21	TRUE

Supplementary Table 5. BMEC Emcn$^+$ / Emcn$^-$ Differential expressions of selected 51 genes. Differential expressions of selected 51 genes related to EC identity markers from bulk RNA-seq analysis of P0 Emcn$^+$ and P0 Emcn$^-$ ECs (n=2).

Supplementary Table 6.

	Arterial EC markers	
	**Gene**	**Reference**
**1**	Ace	Mouse (Xu et al. 2018)
**2**	Cav1	Mouse (Xu et al. 2018)
**3**	Cd200	Mouse (Xu et al. 2018)
**4**	Cd34	Mouse (Xu et al. 2018)
**5**	Dll4	Mouse (Shutter et al. 2000)
**6**	Efnb2	Mouse (Adams et al. 1999; Wang et al. 1998; Xu et al. 2018)
**7**	Fbln2	Mouse (Zhao et al. 2018)
**8**	Jam2	Mouse (Xu et al. 2018)
**9**	Ltbp4	Mouse (Xu et al. 2018)
**10**	Ly6a	Mouse (Xu et al. 2018)
**11**	Notch1	Mouse (Villa et al. 2001)
**12**	Notch4	Mouse (Villa et al. 2001)
**13**	Podxl	Mouse (Xu et al. 2018)
**14**	Serpinf1	Mouse (Xu et al. 2018)
**15**	Sox17	Mouse (Zhao et al. 2018)
**16**	Sox18	Zebrafish (Cermenati et al., 2008; Herpers et al., 2008; Pendeville et al., 2008)
**17**	Sparcl1	Mouse (Xu et al. 2018)
**18**	Timp4	Mouse (Xu et al. 2018)
	**Sinusoidal (capillary) / Venous EC markers**	
	**Gene**	**Reference**
**1**	Abcc9	Mouse (Xu et al. 2018)
**2**	Ap1b1	Mouse (Xu et al. 2018)
**3**	Dnase1l3	Mouse (Xu et al. 2018)
**4**	Emcn	Mouse (Morgan et al. 1999)
**5**	Eng	Mouse (Singh et al. 2020)
**6**	Ephb4	Mouse (Adams et al. 1999; Wang et al. 1998)
**7**	Epor	Mouse (Xu et al. 2018)
**8**	Fabp4	Mouse (Fuseya et al. 2017)
**9**	Flt4	Mouse (Kaipainen et al. 1995; Xu et al. 2018)
**10**	Gpm6a	Mouse (Xu et al. 2018)
**11**	Jag2	Mouse (Sacma et al. 2019)
**12**	Kcnj8	Mouse (Xu et al. 2018)
**13**	Mrc1	Mouse (Xu et al. 2018)
**14**	Nrp2	Mouse (Yuan et al. 2002)
**15**	Sele	Mouse (Xu et al. 2018)
**16**	Selp	Mouse (Xu et al. 2018)
**17**	Stab2	Mouse (Xu et al. 2018)
**18**	Tspan7	Mouse (Xu et al. 2018)
**19**	Ubd	Mouse (Xu et al. 2018)
**20**	Vcam1	Mouse (Zhao et al. 2018; Xu et al. 2018)
	**Tip EC markers**	
	**Gene**	**Reference**
**1**	Angpt2	Human (Siemerink et al. 2012); Mouse (Lee et al. 2014)

	**Tip EC markers (continued)**	
**2**	Apln	Human (Siemerink et al. 2012), Mouse (Lee et al. 2014)
**3**	Cd34	Human (Siemerink et al. 2012; Yetkin-Arik et al. 2019)
**4**	Cldn5	Mouse (Zhao et al. 2018)
**5**	Cotl1	Mouse (Zhao et al. 2018)
**6**	Cxcr4	Human (Siemerink et al. 2012)
**7**	Dll4	Mouse (Zhao et al. 2018; Lee et al. 2014), Human (Siemerink et al. 2012)
**8**	Ednrb	Mouse (Zhao et al. 2018)
**9**	Esm1	Mouse (Lee et al. 2014)
**10**	Flt4	Mouse (Tammela et al. 2008), Human (Siemerink et al. 2012)
**11**	Fscn1	Mouse (Zhao et al. 2018)
**12**	Gpihbp1	Mouse (Zhao et al. 2018)
**13**	Igfbp3	Mouse (Zhao et al. 2018)
**14**	Inhbb	Mouse (Zhao et al. 2018)
**15**	Jup	Mouse (Zhao et al. 2018)
**16**	Kcne3	Mouse (Zhao et al. 2018)
**17**	Kcnj8	Mouse (Zhao et al. 2018)
**18**	Kdr	Mouse (Tammela et al. 2008), Human (Siemerink et al. 2012)
**19**	Marcksl1	Mouse (Zhao et al. 2018)
**20**	Mcam	Mouse (Zhao et al. 2018)
**21**	Mest	Mouse (Zhao et al. 2018)
**22**	N4bp3	Mouse (Zhao et al. 2018)
**23**	Nid2	Mouse (Zhao et al. 2018)
**24**	Notch4	Mouse (Zhao et al. 2018)
**25**	Pdgfb	Human (Siemerink et al. 2012), Mouse (Lee et al. 2014)
**26**	Plxnd1	Mouse (Zhao et al. 2018), Human (Siemerink et al. 2012)
**27**	Sox17	Mouse (Lee et al. 2014)
	**Stalk EC markers**	
	**Gene**	**Reference**
**1**	Adh7	Human (Yetkin-Arik et al. 2019)
**2**	Aldh2	Human (Yetkin-Arik et al. 2019)
**3**	Eno2	Human (Yetkin-Arik et al. 2019)
**4**	Hk2	Human (Yetkin-Arik et al. 2019)
**5**	Il6st	Mouse (Zhao et al. 2018)
**6**	Lgals3	Mouse (Zhao et al. 2018)
**7**	Meox2	Mouse (Zhao et al. 2018)
**8**	Pgm1	Human (Yetkin-Arik et al. 2019)

Supplementary Table 6. EC markers list

List of marker genes for arterial, sinusoidal, tip, and stalk EC and their references.

Supplementary Table 7.

# Mouse (4-6-month-old)	CD45-Ter119-tdT+ # from fresh BM
#1	8,174
#2	3,644
#3	3,313
#4	3,137
#5	4,002
#6	6,255
#7	4,345
#8	4,653
#9	8,818
#10	8,092
#11	7,141
**Mean±SD**	**5,598 ± 2,148**

Supplementary Table 7. Summary of absolute cell numbers of freshly sorted BMEC.
Absolute cell numbers of $CD45^-Ter119^-tdT^+$ BMECs freshly sorted from two legs of 4-6-month-old *Tie2-CreERT2;Rosa26-tdTomato* mice. All numbers were derived from the cell count on the sorter at the end of sorting (n=11).

Supplementary Table 8

# Mouse (4-6-month-old)	CD45-tdT+Emcn+ # from P0 WBM culture (Day 14)	CD45-tdT+Emcn- # from P0 WBM culture (Day 14)
#1	37,264	164,488
#2	36,934	240,172
#3	49,591	663,227
#4	114,860	1,236,180
#5	91,122	832,667
#6	128,359	715,586
#7	54,170	1,242,098
#8	102,201	995,390
#9	47,964	1,182,864
#10	38,042	1,021,794
#11	75,920	541,498
**Mean±SD**	**70,584 ± 33,573**	**803,269 ± 378,225**

Supplementary Table 8. Summary of absolute cell numbers of BMEC sorted from P0 WBM culture. Absolute numbers of $CD45^{-}tdT^{+}Emcn^{+}$ (left column, in blue) and $CD45^{-}tdT^{+}Emcn^{-}$ (right column, in red) BMECs sorted from P0 WBM culture at day 14. Cells for culture were harvested from 4-6 months old *Tie2-CreERT2;Rosa26-tdTomato* mice. All numbers were derived from the cell count on the sorter at the end of sorting sorting (n=11).

Supplementary Table 9. Antibodies

REAGENT or RESOURCE	SOURCE	IDENTIFIER
**Antibodies**		
eFluor 450 anti-mouse CD45, Clone 30-F11<br><br>https://www.thermofisher.com/antibody/product/CD45-Antibody-clone-30-F11-Monoclonal/48-0451-80	eBioscience	Cat#48-0451-82
Endomucin, Clone V.7C7<br><br>https://www.scbt.com/p/endomucin-antibody-v-7c7?requestFrom=search	Santa Cruz	Cat#sc-65495
Endomucin Alexa Fluor 488, Clone V.7C7<br><br>https://www.scbt.com/p/endomucin-antibody-v-7c7?requestFrom=search	Santa Cruz	Cat#sc-65495 AF488
Alexa Fluor 488 anti-mouse CD31<br><br>https://www.rndsystems.com/products/mouse-rat-cd31-pecam-1-alexa-fluor-488-conjugated-antibody_fab3628g	R&D Systems	Cat#FAB3628G
FITC anti-mouse CD31, Clone MEC13.3<br><br>https://www.bdbiosciences.com/en-us/products/reagents/flow-cytometry-reagents/research-reagents/single-color-antibodies-ruo/fitc-rat-anti-mouse-cd31.553372	BD Bioscience	Cat#553372

Continued		
PE-Cy7 anti-mouse CD31, Clone MEC13.3<br><br>https://www.biolegend.com/en-us/products/pe-cyanine7-anti-mouse-cd31-antibody-12996	Biolegend	Cat#102524
PerCP-Cy5.5 anti-mouse Sca-1, Clone D7<br><br>https://www.biolegend.com/en-us/products/percp-cyanine5-5-anti-mouse-ly-6a-e-sca-1-antibody-4285	Biolegend	Cat#108124
PerCP-Cy5.5 anti-mouse Flk-1, Clone AVAS 12α1<br><br>https://www.bdbiosciences.com/en-us/products/reagents/flow-cytometry-reagents/research-reagents/single-color-antibodies-ruo/percp-cy-5-5-rat-anti-mouse-flk-1.560681	BD Bioscience	Cat#560681
PE-Cy7 anti-mouse CD105, Clone MJ7/18<br><br>https://www.biolegend.com/en-us/products/pe-cyanine7-anti-mouse-cd105-antibody-4573	Biolegend	Cat#120410
PE-Cy7 anti-mouse CD140a, Clone APA5<br><br>https://www.thermofisher.com/antibody/product/CD140a-PDGFRA-Antibody-clone-APA5-Monoclonal/25-1401-82	eBioscience	Cat#25-1401-82
APC anti-mouse CD31, Clone MEC13.3<br><br>https://www.biolegend.com/en-us/products/apc-anti-mouse-cd31-antibody-375	Biolegend	Cat#102510

Continued		
APC anti-mouse CD45, Clone 30-F11<br><br>https://www.bdbiosciences.com/en-us/products/reagents/flow-cytometry-reagents/research-reagents/single-color-antibodies-ruo/apc-rat-anti-mouse-cd45.559864	BD Bioscience	Cat#559864
Alexa Fluor 488 anti-mouse CD144, Clone BV13<br><br>https://www.thermofisher.com/antibody/product/CD144-VE-cadherin-Antibody-clone-eBioBV13-BV13-Monoclonal/53-1441-82	eBioscience	Cat#53-1441-82
APC anti-mouse CD144, Clone BV13<br><br>https://www.biolegend.com/en-us/products/apc-anti-mouse-cd144-ve-cadherin-antibody-6989	Biolegend	Cat#138011
APC anti-mouse F4/80, Clone BM8<br><br>https://www.thermofisher.com/antibody/product/F4-80-Antibody-clone-BM8-Monoclonal/MF48005	Invitrogen	Cat#MF48005
APC-eFluor 780 anti-mouse c-Kit, Clone 2B8<br><br>https://www.thermofisher.com/antibody/product/CD117-c-Kit-Antibody-clone-2B8-Monoclonal/47-1171-82	eBioscience	Cat#47-1171-82
APC-Cy7 anti-mouse CD11b, Clone M1/70<br><br>https://www.biolegend.com/en-us/products/apc-cyanine7-anti-mouse-human-cd11b-antibody-3930	Biolegend	Cat#101226
FITC anti-mouse CD3ε, Clone 145-2C11<br><br>https://www.biolegend.com/en-us/products/fitc-anti-mouse-cd3epsilon-antibody-23	Biolegend	Cat#100306
FITC anti-mouse CD4, Clone RM4-5<br><br>https://www.biolegend.com/en-us/products/fitc-anti-mouse-cd4-antibody-480	Biolegend	Cat#100510

Continued		
FITC anti-mouse CD8a, Clone 53-6.7 https://www.biolegend.com/en-us/products/fitc-anti-mouse-cd8a-antibody-153	Biolegend	Cat#100706
FITC anti-mouse CD11b, Clone M1/70 https://www.biolegend.com/en-us/products/fitc-anti-mouse-human-cd11b-antibody-347	Biolegend	Cat#101206
FITC anti-mouse B220, Clone RA3-6B2 https://www.biolegend.com/en-us/products/fitc-anti-mouse-human-cd45r-b220-antibody-445	Biolegend	Cat#103206
FITC anti-mouse Gr-1, Clone RB6-8C5 https://www.bdbiosciences.com/en-us/products/reagents/flow-cytometry-reagents/research-reagents/single-color-antibodies-ruo/fitc-rat-anti-mouse-ly-6g-and-ly-6c.553127	BD Bioscience	Cat#553127
FITC anti-mouse Ter119, Clone TER-119 https://www.biolegend.com/en-us/products/fitc-anti-mouse-ter-119-erythroid-cells-antibody-1865	Biolegend	Cat#116206
PerCP-Cy5.5 anti-mouse B220, Clone RA3-6B2 https://www.biolegend.com/en-us/products/percp-cyanine5-5-anti-mouse-human-cd45r-b220-antibody-4267	Biolegend	Cat#103236
PE-Cy7 anti-mouse CD3ε, Clone 145-2C11 https://www.biolegend.com/en-us/products/pe-cyanine7-anti-mouse-cd3epsilon-antibody-1899	Biolegend	Cat#100320
Goat anti-Rat IgG (H+L) Secondary Antibody, Alexa Fluor 488 https://www.thermofisher.com/antibody/product/Goat-anti-Rat-IgG-H-L-Cross-Adsorbed-Secondary-Antibody-Polyclonal/A-11006	Invitrogen	Cat#A11006
Donkey anti-Rat IgG (H+L) Secondary Antibody, Alexa Fluor 488 https://www.thermofisher.com/antibody/product/Donkey-anti-Rat-IgG-H-L-Highly-Cross-Adsorbed-Secondary-Antibody-Polyclonal/A-21208	Invitrogen	Cat#A21208
**Chemicals and Recombinant Proteins**		
Tamoxifen	Sigma-Aldrich	Cat#T5648-1G
Sunflower seed oil	Sigma-Aldrich	Cat#S5007-250ML
Collagenase Type I	Worthington	Cat#LS004196
BSA	Fisher Scientific	Cat#BP1600-100
DNase I, grade II, from bovine pancreas	Roche	Cat#10104159001
DAPI	Invitrogen	Cat#D3571

Continued		
EDTA	Fisher Scientific	Cat#BP2482-500
VECTASHIELD Antifade Mounting Medium	Vector Laboratories	Cat#H-1000-10
Recombinant Murine SCF	Peprotech	Cat#250-03
iScript™ cDNA Synthesis Kit	BioRad	Cat#170-8891
Corning™ Matrigel™ GFR Membrane Matrix	Corning	Cat#356231
Corning™ Matrigel™ Basement Membrane Matrix	Corning	Cat#356237
EasySep™ Mouse FITC Positive Selection Kit II	STEMCELL Technologies	Cat#17668
EasySep™ Mouse Hematopoietic Progenitor Cell Isolation Kit	STEMCELL Technologies	Cat#19856
TRIzol Reagent	Invitrogen	Cat#15596026
MesenCult Expansion Kit (Mouse)	STEMCELL Technologies	Cat#05514, 05515, and 05500
EGM-2 Endothelial Cell Growth Medium-2 BulletKit	Lonza	Cat#CC-3156 & CC-4176
**Software**		
Photoshop	Adobe	2021
Graphpad Prism	GraphPad	9
Microsoft Excel	Microsoft	2103
FACS Diva	BD	8.02
FlowJo	BD	10.7.2
ImageJ	NIH	1.53e
ZEN	Zeiss	3.1

Supplementary Table 10. Primers

Gene	Forward	Reverse
18s	cgattggatggtttagtgagg	agttcgaccgtccttctcagc
Ace	cgaccctttactggttgtctt	gcttccttcccctctgttt
Acss2	tgcctctactgctttgttacct	gagatggttgatgacagatgg
Alpl	ctgatgtggaatacgaactgg	catagtgggaatgcttgtgtc
Angpt2	cccgtaagtttgctactgttatg	aggctgtgtgaaaggagatg
Apln	tattcaggagggcattctttt	gcatagcgagtctttttgtga
Bglap	ggaccatctttctgctcactct	taccttattgccctcctgctt
Cd34	cccctactttatggaaaccaa	tcaggaacaggtgaacaaga
Cdh5	tgaagaaagaagaggcagaca	ccagtcgttgaggaagtca
Cxcr4	ttgtccacgccaccaacagtca	tgaaacaccaccatccacaggc
Dll1	cttctttcgcgtatgcctcaa	catcaggcaggctgaagga
Dll4	tgcctgaacagaggtccaag	cattgaagcagggtccggag
Efnb2	gtgtatgagtgggtgtgtggt	gaggttagggtgatggaaaga
Emcn	cagtatcattttgcctgtggt	catttccgttttctggagtg
Eno2	gcttggttgaggctttagtgt	ttacaggagatggctgactga
Ephb4	ggattgttggcagaaggac	accgaaagcagaatagtgagg
Epor	gacctggaccctctcatctt	agaccctcaaactcgctctc
Esm1	aggcgataaaacaagaccaga	aaccagagatgagaagtgatgg
Fbln2	ctaagtggctttttgctgtga	gtggatgaaggaatggaatgt
Flt4	aaagcaggcatagaccagaag	acctccccatactcgttgtt
Gapdh	aagcccatcaccatcttcca	tagactccacgacatactca
Hk2	acaccaatgtcctgttctctg	gacccccgtatcttgtttatt
Ibsp	aaaatggagacggcgatagtt	acccgagagtgtggaaagtg
Il6st	gcagtttcaccttttactctca	actttcaccatctcccttcac
Kdr	cccattgagtccaactacaca	cattcacaaccagagagacca
Ltbp4	ctccttccgctgtatctgtc	cctctgtgtctgtccatcct
Ly6a	ctccacccttgtccttttatc	agcacctacctacccagca
Meox2	cattctcaggaaagcaccatt	acatccatctccctctctgtc
Mrc1	cctggcaagattagagaaaca	agggttgacatgagacctacc
Pdgfb	agactggaggaactctgatgg	cgtaggggaagtggaaaga
Pecam1	gacagacccttccaccaag	gatgaccactccaatgacaac
Ptprc (Cd45)	gcctacactctccgaactttt	gcttgtgatacttcatcccttc
Selp	cactttcccacctctccac	gtaacaacaagccacagacca
Serpinf1	gattttcatttggacgaggac	agttctgggtcacggtcag
Sox17	agaaaccctaaacacaaacagc	tggaagaaaagacaaacagga
Sparcl1	ccccaacaaggataagcac	taaagcaggtgaggtggatg
Spi1	agaagctgatggcttggagc	gcgaatctttttcttgctgcc
Stab2	atgtccttcccctcactcac	cttatttttcggtagcccact
