# Supplementary material for: Integration of single-cell transcriptomes and biological function reveals distinct behavioral patterns in bone marrow endothelium
Source: Nat Commun. 2022 Nov 24;13:7235. doi: 10.1038/s41467-022-34425-z (PMC9700769; doi:10.1038/s41467-022-34425-z)
Supplement: Supplementary file 3 — Description of Additional Supplementary Files [file 41467_2022_34425_MOESM3_ESM.pdf]

Description of Additional Supplementary Files

File Name: Supplementary Movie 1
Description: **Time-lapse live imaging of P0 WBM culture from day 5 to day 13: distinct self-organization of two BMEC populations.** Images were taken every 45 minutes and all connected after all.

File Name: Supplementary Movie 2
Description: **Serial snapshot images of P0 WBM culture from day 2 to day 11: from single cell to colony.**
Video focuses on a colony formation starting from one single cell. Images were taken every and all connected after all.

File Name: Supplementary Movie 3
Description: **Time-lapse live imaging of cords-networks formation in Matrigel by BMEC Emcn- at different passages.** Images were taken every 45 minutes and all connected at the end.
