# Supplementary material for: Integration of single-cell transcriptomes and biological function reveals distinct behavioral patterns in bone marrow endothelium
Source: Nat Commun. 2022 Nov 24;13:7235. doi: 10.1038/s41467-022-34425-z (PMC9700769; doi:10.1038/s41467-022-34425-z)
Supplement: Supplementary file 7 — Reporting Summary [file 41467_2022_34425_MOESM7_ESM.pdf]

nature portfolio

Corresponding author(s): Nadia Carlesso and Young-Woong Kim
DBPR NCOMMS-22-11610

Last updated by author(s): 09-30-2022

Reporting Summary

Nature Portfolio wishes to improve the reproducibility of the work that we publish. This form provides structure for consistency and transparency in reporting. For further information on Nature Portfolio policies, see our Editorial Policies and the Editorial Policy Checklist.

Statistics

For all statistical analyses, confirm that the following items are present in the figure legend, table legend, main text, or Methods section.

n/a	Confirmed	
☐	☒	The exact sample size (*n*) for each experimental group/condition, given as a discrete number and unit of measurement
☐	☒	A statement on whether measurements were taken from distinct samples or whether the same sample was measured repeatedly
☐	☒	The statistical test(s) used AND whether they are one- or two-sided<br>*Only common tests should be described solely by name; describe more complex techniques in the Methods section.*
☒	☐	A description of all covariates tested
☒	☐	A description of any assumptions or corrections, such as tests of normality and adjustment for multiple comparisons
☐	☒	A full description of the statistical parameters including central tendency (e.g. means) or other basic estimates (e.g. regression coefficient) AND variation (e.g. standard deviation) or associated estimates of uncertainty (e.g. confidence intervals)
☐	☒	For null hypothesis testing, the test statistic (e.g. *F*, *t*, *r*) with confidence intervals, effect sizes, degrees of freedom and *P* value noted<br>*Give P values as exact values whenever suitable.*
☒	☐	For Bayesian analysis, information on the choice of priors and Markov chain Monte Carlo settings
☒	☐	For hierarchical and complex designs, identification of the appropriate level for tests and full reporting of outcomes
☒	☐	Estimates of effect sizes (e.g. Cohen's *d*, Pearson's *r*), indicating how they were calculated

Our web collection on statistics for biologists contains articles on many of the points above.

Software and code

Policy information about availability of computer code

Data collection	ZEN (Zeiss), FACS Diva (BD)
Data analysis	ZEN (Zeiss), FlowJo (BD), ImageJ (NIH), Excel (Microsoft), Prism (GraphPad), Photoshop (Adobe); Cell Ranger v5.0.0, 10X Genomics; nf-core RNASeq pipeline, v1.4.2; STAR, v2.7.10a; Salmon, v1.5.2; Base R, v4.1.3; tximeta R package, v1.8.0; DESeq2 R package, v1.28.0; Seurat R package, v4.0.5; clusterProfiler R package, v4.2.0; scran R package, v1.22.1

For manuscripts utilizing custom algorithms or software that are central to the research but not yet described in published literature, software must be made available to editors and reviewers. We strongly encourage code deposition in a community repository (e.g. GitHub). See the Nature Portfolio guidelines for submitting code & software for further information.

Data

Policy information about availability of data

All manuscripts must include a data availability statement. This statement should provide the following information, where applicable:

- Accession codes, unique identifiers, or web links for publicly available datasets
- A description of any restrictions on data availability
- For clinical datasets or third party data, please ensure that the statement adheres to our policy

The scRNA-seq and bulk raw data generated in this study have been deposited in the Gene Expression Omnibus database under the accession number GSE206977

[https://www.ncbi.nlm.nih.gov/geo/query/acc.cgi?acc=GSE206977].
The source data underlying Fig. 2b, 3d-f, 4a-c, 4e-f, 5c-g, 6c, 6e-f, 7d, 7g, and Supplementary Fig. 1a, 2e, 3a-b, 3d-f, 3h, 4a-c, 6a-d are provided as a Source Data file.

Human research participants

Policy information about studies involving human research participants and Sex and Gender in Research.

Reporting on sex and gender	No human studies were performed
Population characteristics	N/A
Recruitment	N/A
Ethics oversight	N/A

Note that full information on the approval of the study protocol must also be provided in the manuscript.

Field-specific reporting

Please select the one below that is the best fit for your research. If you are not sure, read the appropriate sections before making your selection.

☒ Life sciences ☐ Behavioural & social sciences ☐ Ecological, evolutionary & environmental sciences

For a reference copy of the document with all sections, see nature.com/documents/nr-reporting-summary-flat.pdf

Life sciences study design

All studies must disclose on these points even when the disclosure is negative.

Sample size

For estimating sample size used (2-6), we performed power analysis with effect size, "Cohen's d" (Ref. 1), which was predicted with previous experience/data. For some experiments, small number of sample size (n=2) was used (as an example, data from Figure 6f). This is because we already knew that the effect size is large (d = 5.03; PPT Slide#2) enough to provide high statistical power (91.8%; PPT Slide#2) when two independent means were compared with one-side significance level (≤ 0.05).
For estimating effect size and power analysis for sample size, we used R packages, "effectsize" (Ref. 2) and "pwr" (Ref. 3), respectively.
References
1. Cohen, J. (1988) Statistical power analysis for the behavioral sciences (2nd ed.).Hillsdale, NJ: Lawrence Erlbaum.
2. Ben-Shachar M, Lüdecke D, Makowski D (2020). effectsize: Estimation of Effect Size Indices and Standardized Parameters. Journal of Open Source Software, 5(56), 2815. doi: 10.21105/joss.02815
3. R package, pwr, Champely, Stephane. 2020. Pwr: Basic Functions for Power Analysis. https://CRAN.R-project.org/package=pwr.

Data exclusions

No data were excluded from the analysis.

Replication

The majority of experiments were replicated on average 5 independent times (range 3-12). We have conducted 2 independent experiments only in 3 out of the 132 experiments conducted. These experiments were supported by complementary approaches, and provided concordant results. The precise number is stated in each figure legend.
For primary cell cultures, independent experiments refer to fully independent cultures starting from different animal. Bulk and single-cell RNA-seq experiments were replicated using at least 2 independent biological samples per condition.

Randomization

Based on their genotype, randomized cohorts including both male and female animals were distributed in an unblinded manner into the experimental time points for analysis.

Blinding

This study focused on establishing a protocol for BM-EC culture and characterizatio: experiments aimed at validate the robustness of the method were repeated by three different lab members independently, and lead to the same results.

Reporting for specific materials, systems and methods

We require information from authors about some types of materials, experimental systems and methods used in many studies. Here, indicate whether each material, system or method listed is relevant to your study. If you are not sure if a list item applies to your research, read the appropriate section before selecting a response.

Materials & experimental systems

n/a	Involved in the study	
☐	☒	Antibodies
☒	☐	Eukaryotic cell lines
☒	☐	Palaeontology and archaeology
☐	☒	Animals and other organisms
☒	☐	Clinical data
☒	☐	Dual use research of concern

Methods

n/a	Involved in the study	
☒	☐	ChIP-seq
☐	☒	Flow cytometry
☒	☐	MRI-based neuroimaging

Antibodies

Antibodies used

eFluor 450 anti-mouse CD45, Clone 30-F11 (eBioscience, Cat#48-0451-82); Endomucin, Clone V.7C7 (Santa Cruz, Cat#sc-65495); Endomucin Alexa Fluor 488, Clone V.7C7 (Santa Cruz, Cat#sc-65495 AF488); Alexa Fluor 488 anti-mouse CD31 (R&D Systems, Cat#FAB3628G); FITC anti-mouse CD31, Clone MEC13.3 (BD Bioscience, Cat#553372); PE-Cy7 anti-mouse CD31, Clone MEC13.3 (Biolegend, Cat#102524); PerCP-Cy5.5 anti-mouse Sca-1, Clone D7 (Biolegend, Cat#108124); PerCP-Cy5.5 anti-mouse Flk-1, Clone AVAS 12a1 (BD Bioscience, Cat#560681); PE-Cy7 anti-mouse CD105, Clone MJ7/18 (Biolegend, Cat#120410); PE-Cy7 anti-mouse CD140a, Clone APA5 (eBioscience, Cat#25-1401-82); APC anti-mouse CD31, Clone MEC13.3 (Biolegend, Cat#102510); APC anti-mouse CD45, Clone 30-F11 (BD Bioscience, Cat#559864); Alexa Fluor 488 anti-mouse CD144, Clone BV13 (eBioscience, Cat#53-1441-82); APC anti-mouse CD144, Clone BV13 (Biolegend, Cat#138011); APC anti-mouse F4/80, Clone BM8 (Invitrogen, Cat#MF48005); APC-eFluor 780 anti-mouse c-Kit, Clone 2B8 (eBioscience, Cat#47-1171-82); APC-Cy7 anti-mouse CD11b, Clone M1/70 (Biolegend, Cat#101226); FITC anti-mouse CD3e, Clone 145-2C11 (Biolegend, Cat#100306); FITC anti-mouse CD4, Clone RM4-5 (Biolegend, Cat#100510); FITC anti-mouse CD8a, Clone 53-6.7 (Biolegend, Cat#100706); FITC anti-mouse CD11b, Clone M1/70 (Biolegend, Cat#101206); FITC anti-mouse B220, Clone RA3-6B2 (Biolegend, Cat#103206); FITC anti-mouse Gr-1, Clone RB6-8C5 (BD Bioscience, Cat#553127); FITC anti-mouse Ter119, Clone TER-119 (Biolegend, Cat#116206); PerCP-Cy5.5 anti-mouse B220, Clone RA3-6B2 (Biolegend, Cat#103236); PE-Cy7 anti-mouse CD3e, Clone 145-2C11 (Biolegend, Cat#100320); Goat anti-Rat IgG (H+L) Secondary Antibody, Alexa Fluor 488 (Invitrogen, Cat#A11006); Donkey anti-Rat IgG (H+L) Secondary Antibody, Alexa Fluor 488 (Invitrogen, Cat#A21208).

For flow cytometry, all antibodies were used with 1 uL / 1 x 10^7 cells. For immunostaining, all primary antibodies for Emcn, CD31, Sma, VE-Cadherin, and Laminin were used at 1:100 dilution, and all secondary antibodies at 1:400 dilution.

Validation

https://www.thermofisher.com/antibody/product/CD45-Antibody-clone-30-F11-Monoclonal/48-0451-80;
https://www.scbt.com/p/endomucin-antibody-v-7c7 requestFrom=search;
https://www.rndsystems.com/products/mouse-rat-cd31-pecam-1-alexa-fluor-488-conjugated-antibody_fab3628g;
https://www.bdbiosciences.com/en-us/products/reagents/flow-cytometry-reagents/research-reagents/single-color-antibodies-ruo/fitc-rat-anti-mouse-cd31.553372;
https://www.biolegend.com/en-us/products/pe-cyanine7-anti-mouse-cd31-antibody-12996;
https://www.biolegend.com/en-us/products/percp-cyanine5-5-anti-mouse-ly-6a-e-sca-1-antibody-4285;
https://www.bdbiosciences.com/en-us/products/reagents/flow-cytometry-reagents/research-reagents/single-color-antibodies-ruo/percp-cy-5-5-rat-anti-mouse-flk-1.560681;
https://www.biolegend.com/en-us/products/pe-cyanine7-anti-mouse-cd105-antibody-4573;
https://www.thermofisher.com/antibody/product/CD140a-PDGFRA-Antibody-clone-APA5-Monoclonal/25-1401-82;
https://www.biolegend.com/en-us/products/apc-anti-mouse-cd31-antibody-375;
https://www.bdbiosciences.com/en-us/products/reagents/flow-cytometry-reagents/research-reagents/single-color-antibodies-ruo/apc-rat-anti-mouse-cd45.559864;
https://www.thermofisher.com/antibody/product/CD144-VE-cadherin-Antibody-clone-eBioBV13-BV13-Monoclonal/53-1441-82;
https://www.biolegend.com/en-us/products/apc-anti-mouse-cd144-ve-cadherin-antibody-6989;
https://www.thermofisher.com/antibody/product/F4-80-Antibody-clone-BM8-Monoclonal/MF48005;
https://www.thermofisher.com/antibody/product/CD117-c-Kit-Antibody-clone-2B8-Monoclonal/47-1171-82;
https://www.biolegend.com/en-us/products/apc-cyanine7-anti-mouse-human-cd11b-antibody-3930;
https://www.biolegend.com/en-us/products/fitc-anti-mouse-cd3epsilon-antibody-23;
https://www.biolegend.com/en-us/products/fitc-anti-mouse-cd4-antibody-480;
https://www.biolegend.com/en-us/products/fitc-anti-mouse-cd8a-antibody-153;
https://www.biolegend.com/en-us/products/fitc-anti-mouse-human-cd11b-antibody-347;
https://www.biolegend.com/en-us/products/fitc-anti-mouse-human-cd45r-b220-antibody-445;
https://www.bdbiosciences.com/en-us/products/reagents/flow-cytometry-reagents/research-reagents/single-color-antibodies-ruo/fitc-rat-anti-mouse-ly-6g-and-ly-6c.553127;
https://www.biolegend.com/en-us/products/fitc-anti-mouse-ter-119-erythroid-cells-antibody-1865;
https://www.biolegend.com/en-us/products/percp-cyanine5-5-anti-mouse-human-cd45r-b220-antibody-4267;
https://www.biolegend.com/en-us/products/pe-cyanine7-anti-mouse-cd3epsilon-antibody-1899;
https://www.thermofisher.com/antibody/product/Goat-anti-Rat-IgG-H-L-Cross-Adsorbed-Secondary-Antibody-Polyclonal/A-11006;
https://www.thermofisher.com/antibody/product/Donkey-anti-Rat-IgG-H-L-Highly-Cross-Adsorbed-Secondary-Antibody-Polyclonal/A-21208.

Animals and other research organisms

Policy information about studies involving animals; ARRIVE guidelines recommended for reporting animal research, and Sex and Gender in Research

Laboratory animals	Tie2-CreERT2;Rosa26-tdTomato and Osx-Cre;Rosa26-tdTomato mice were used in the study.<br>We did not find differences in obtaining EC from adult mice ranging from 2-months to 7-months of age. Majority (>80%) of the experiments were performed with mice 4-6 months old.<br>We did not observed sex-associated differences in the isolation and characteristics of BM -derived EC. Thus, animals were used regardless of their sex.<br><br>Tie2-CreERT2;Rosa26-tdTomato mice:<br>Tie2-CreERT2 mice express a tamoxifen-inducible Cre recombinase gene under direction of the TEK receptor tyrosine kinase (Tek) promoter on a bacterial artificial chromosome (BAC) transgene. This specific strain obtained from Dr. Yi Zheng, was developed by using the DNA fragment containing the intron sequence of the -globulin gene, a Cre recombinase, the mutated ligand binding domain of the estrogen receptor, and SV40 polyA (klenow enzyme filled Stu I site- Xba I DNA fragment) excised from plasmid pCreERT2 and inserted into the EcoR V-Xba I site of plasmid pBSTie2-2. The resulting plasmid was called pTie2P-CreERT2. The Sal I fragment containing Tie2 promoter and CreERT2 was excised from pTie2P-CreERT2 and cloned upstream into SV40 polyA signal and the first intron sequence of pBSpolyATie2-10 to generate the transgenic construct. The final Tie2-CreERT2 transgene, comprising the Tie2 promoter, β-globulin intron sequence, CreERT2 fusion gene, 2 polyadenylation signal (pA) fragments, and Tie2 enhancer sequence was excised from the vector and used for C57BL/6 pronuclear micro-injection to generate the transgenic mouse line. The Cre-ERT2 protein is expressed in the cytoplasm of endothelial cells and can only access the nuclear compartment upon exposure to tamoxifen. We crossed these mice with a Rosa26-tdTomato strain (B6;129S6-Gt(ROSA)26Sortm9(CAG-tdTomato)Hze/J; Ai9 or Ai9 (RCL-tdT); Jackson Laboratory stock number: 007905) to generate animals expressing tdTomato (tdT) fluorescence under the control of the Tie2-Cre promoter, to mark endothelial cells and delineate the vasculature.<br><br>Osx-Cre;Rosa26-tdTomato mice:<br>Osx-Cre mice were generated by pronuclear injection of a bacterial artificial chromosome (BAC) containing the Osx1 gene targeted at exon 1, using standard BAC recombination. This specific strain was obtained from Dr. Ernestina Schipani and was generated as described by Nakashima K. et al. 2002. We crossed these mice with a Rosa26-tdTomato reporter strain (B6;129S6-Gt(ROSA)26Sortm9(CAG-tdTomato)Hze/J; Ai9 or Ai9 (RCL-tdT); Jackson Laboratory stock number: 007905) to generate animals expressing a robust tdT fluorescence under the control of the Osx-Cre promoter, hence only in bone marrow osteolineage cells. In our study this mouse strain was only used as a source of sorted BM tdT+ MSC to compare their transcriptome profiles with the transcriptome of BM tdT+ BMEC.
Wild animals	No wild animals were used in this study.
Reporting on sex	For all of our experiments, there was no sex dependency.
Field-collected samples	No field-collected samples were used in this study.
Ethics oversight	All mice were group housed under specific pathogen-free conditions. Animal experiments were performed when mice were 1.5-7 months old using protocols approved by City of Hope Animal Care and Use Committee.

Note that full information on the approval of the study protocol must also be provided in the manuscript.

Flow Cytometry

Plots

Confirm that:

- [x] The axis labels state the marker and fluorochrome used (e.g. CD4-FITC).
- [x] The axis scales are clearly visible. Include numbers along axes only for bottom left plot of group (a 'group' is an analysis of identical markers).
- [x] All plots are contour plots with outliers or pseudocolor plots.
- [x] A numerical value for number of cells or percentage (with statistics) is provided.

Methodology

Sample preparation	Flow Cytometry and Cell Sorting<br>At day 14, P0 cultured cells were detached by Trypsin-EDTA (0.05% trypsin, 0.02% EDTA) for 5 minutes and further detached by pipetting. Cells were then suspended in PBS containing 2% FBS at 1 × 10^7 cells/mL and stained for 30 minutes on ice with the following antibodies: APC-anti-CD45, APC-anti-Ter119, and Alexa-488-anti-Emcn. DAPI-CD45-Ter119-tdTomato+ cells from fresh BM and the following populations from P0 cultured cells: DAPI-CD45-tdTomato+Emcn+, DAPI-CD45-tdTomato+Emcn-, DAPI-CD45-tdTomato-, and DAPI-CD45+tdTomato- were sorted by FACSAria Fusion (BD Biosciences). Complete characterization of BMECs was performed by using the following antibodies: CD45, Ter119, CD31, CD144, CD140a,

	CD105, Sca-1, Emcn, Flk-1, CD14, F4/80, and CD11b. Cells were acquired by LSRII (BD Biosciences) and analyzed with FlowJo. For LSK sorting, DAPI-Lin-Sca-1+cKit+ cells from lineage-depleted fresh BM cells using EasySep™ Mouse Hematopoietic Progenitor Cell Isolation Kit (STEMCELL) were sorted by FACSAria Fusion (BD Biosciences).
Instrument	Flow Cytometry studies were performed by LSRII (BD Biosciences). Description: 5 Laser (355nm, 405nm, 488nm, 561nm & 640nm) up to 15 fluorescent parameters.<br><br>Cell sorting was performed by FACSAria Fusion (BD Biosciences). Description: 5 Laser (355nm, 405nm, 488nm, 561nm & 640nm) up to 18 fluorescent parameters. 1-4 Way Sorting into tubes (12X75mm, 1.5ul); 1-2 Way sorting into 15ml tubes. 96/384 Single Cell Deposition. Within a Class II BSC. Temperature control of Sample and Sort product.
Software	FACS Diva (BD) 8.02
Cell population abundance	On average, the % of fresh BMECs (CD45-Ter119-tdT+ cells) was 0.0116 ± 0.0054/mouse; Supplementary Table 1). On average, the % of BMECs after P0 WBM culture was 18.84 ± 6.99/mouse (Supplementary Table 3).
Gating strategy	tdT+ cells were gated from CD45 negative, which was from DAPI negative for sorting. Emcn+ cells were gated from tdT+ cells.

☒ Tick this box to confirm that a figure exemplifying the gating strategy is provided in the Supplementary Information.
